# In Vitro Culture of *Rosmarinus officinalis* L. in a Temporary Immersion System: Influence of Two Phytohormones on Plant Growth and Carnosol Production

**DOI:** 10.3390/ph14080747

**Published:** 2021-07-29

**Authors:** Eder Villegas-Sánchez, Mariana Macías-Alonso, Soraya Osegueda-Robles, Lisset Herrera-Isidrón, Hector Nuñez-Palenius, Joaquín González-Marrero

**Affiliations:** 1 Instituto Politécnico Nacional, UPIIG, Av. Mineral de Valenciana, No. 200, Col. Fracc, Industrial Puerto Interior, Silao 36275, Mexico; e.d.ervillegas@hotmail.com (E.V.-S.); mmacias@ipn.mx (M.M.-A.); moseguedar@ipn.mx (S.O.-R.); lherrerai@ipn.mx (L.H.-I.); 2 División de Ciencias de la Vida, Campus Irapuato-Salamanca, Universidad de Guanajuato, Ex-Hacienda El Copal. Km. 9 Carr. Irapuato-Silao, Irapuato 36824, Mexico; palenius@ugto.mx

**Keywords:** infectious diseases, carnosol, in vitro, cultures, *Rosmarinus officinalis*, rosemary, quantification, temporary immersion system, 6-benzylaminopurine

## Abstract

Emerging infectious diseases have become a major global problem with public health and economic consequences. It is an urgent need to develop new anti-infective therapies. The natural diterpene carnosol exhibit a wide variety of interesting antibacterial and antiviral properties, and it is considered a theoretical inhibitor of COVID-19 M^pro^. However, this compound is present in the family Lamiaceae in low quantities. To obtain carnosol in concentrations high enough to develop pharmacological studies, we evaluated the efficiency of a micropropagation protocol of *Rosmarinus officinalis* using a solid medium and a temporary immersion system (TIS), as well as the effect of 6-benzylaminopurine (6-BAP) and α-naphthaleneacetic acid (NAA) on the growth of shoots. Moreover, we developed and validated an analytical method to quantify carnosol using the H-point standard additions method in the high-performance liquid chromatography diode array detector (HPLC-DAD). After 30 days of culture, TIS produced the maximum number of shoots per explant (24.33 ± 1.15) on a liquid medium supplemented with 6-BAP at 5.0 mg L^−1^. Next, we also evaluated the effect of immersion time and frequency for TIS. After 72 days of culture, the best results were obtained with an immersion cycle of 1 min every 12 h, yielding 170.33 ± 29.40 shoots. The quantification of carnosol on the samples was performed at a flow rate of 1.2 mL min^−1^ using binary isocratic mobile phase system 60:40 (*v*/*v*) 10 mM formic acid (pH 3.0) (A) and acetonitrile (B) on a reverse-phase column. The content of carnosol in the in vitro cultures was around 8-fold higher than in the wild plant. The present study represents an efficient alternative method to obtain carnosol for its pre-clinical and clinical development.

## 1. Introduction

According to the World Health Organization (WHO), the infectious diseases cause more than 5 million deaths globally every year. A clear example is the impact on global health and economy of the novel SARS-CoV-2 virus [1].

Although we have drugs to treat these infectious diseases, it is necessary to develop new therapies to fight these infections and minimize the risk of resistance [2,3].

*Rosmarinus officinalis* L. is a medicinal plant used since ancient times, whose biological properties are attributable to its high content in phytochemicals as carnosol, carnosic acid, rosmarinic acid, rosmanol or ursolic acid, among others [4]. Of particular interests are carnosic acid and its oxidized derivatives, exhibiting various interesting biological activities [5,6].

The presence of both carnosic acid and carnosol is essential for the antibacterial activity of *R. officinalis*, [7]. For example, both compounds enhance the antimicrobial activity of various commercial antibiotics [8,9,10,11,12], they inhibit human immunodeficiency virus type I (HIV-1) [13,14], or the replication and the initial infection of both A- and B-type human respiratory syncytial virus (RSV) [15]. Recently, different theoretical studies revealed that carnosic acid, carnosol and rosmanol showed a strong and stable binding affinity with catalytic site residues of the coronavirus’s main protease (SARS-CoV-2 M^pro^), which plays an essential role during the virus propagation [16,17,18].

Although all the available information suggest that these abietane diterpenes are very promising antiviral and antimicrobial agents to treat present or future infectious diseases such as COVID-19, the main problem for the clinical applications of these compounds it is that they are very expensive compounds obtained from natural sources. To solve this problem, we think that the in vitro culture of *R. officinalis* cells is an alternative to obtain these secondary metabolites in abundance and high purity.. For these reasons, we felt that the Temporary Immersion System (TIS) was the most promising propagation system for the scaling-up of in vitro *R. officinalis*. Liquid culture in TIS allows temporary contact between the plant’s tissue and the liquid medium, improving the supply of nutrients and gas transfers and reducing physiological disorders and plant propagation time. The importance of TIS is evident when verifying its use in the production of several crop and medicinal plants [19].

The culture medium is one of the most critical factors which contribute to successful micropropagation. It is particularly crucial the supplement with plant hormones which regulates plant growth and development. As for growth hormones, cytokinins and auxins are the most commonly used. Among the cytokinins, 6-benzylaminopurine (6-BAP) is one of the most used because it regulates cell division in plant shoots. 1-naphthaleneacetic acid (NAA), an auxin, plays a fundamental role in plant development. Combining these two hormones is widespread, and their interaction often favours plant multiplication and growth in vitro [20]. It is important to note that, in a previous report, Darwesh and Alayafi obtened hight levels of phenolic compounds (10.45 mg g^−1^) in the in vitro culture of *R. officinalis* with 6-BAP at the concentration of 3 mg L^−1^ [21]. Caruso et al. confirmed the presence of carnosic acid in regenerated shoots of this plant cultured on a nutrient medium supplemented with zeatin and indoleacetic acid [22]. Following this previous report, Pérez-Mendoza et al. [23] observed the production of carnosic acid and carnosol in rosemary callus cultured on a medium supplemented with 2,4-dichlorophenoxyacetic acid (2,4-D) and 6-BAP.

In the present manuscript, we describe the results obtained on the evaluation of the phytohormones 6-BAP and NAA on callus proliferation of *R. officinalis* produced in a temporary immersion bioreactor. In addition, we develop a very fast and high-resolution separation system by high-performance liquid chromatography, connected to a diode array detector (HPLC-DAD) using reverse phase, and a sensitive and selective analytical method to quantify carnosol by using H-point standard additions method in HPLC-DAD.

## 2. Results and Discussion

### 2.1. Evaluation of the Different Culture Systems

It was decided to compare the number of shoots per explant obtained after 30 days with two different culture systems, solid media and temporary immersion system (TIS) (Table 1). The results showed significant differences between both culture systems when they were supplemented with the same plant growth regulators (PGR). The TIS system achieved the best results (Table 1, entries 4 and 6). Furthermore, it can be observed that for the same culture systems, the use of 6-BAP provided better results than those obtained with NAA (Table 1, entries 4 and 12). Finally, TIS culture system regenerated the maximum number of shoots on a liquid medium supplemented with 6-BAP at 5.0 mg L^−1^ (24.33 ± 1.15 shoots per explant; Table 1, entry 6).

The number of shoots was higher than that reported by Gabor-Potor & Pop [24], who induced only 16 shoots with 2 mg L^−1^ of 6-BAP combined with 1 mg L^−1^ indole-3-acetic acid (IAA) in solid medium. The same result was observed in the treatments in a liquid medium in comparison to the one obtained by Misra [25], who supplemented the culture medium with immersion frequencies, 0.5 mg L^−1^ IAA and 1.0 mg L^−1^ 2,4-dichlorophenoxyacetic acid (2,4-D). In this study, the greatest number of shoots per explant of *R. officinalis* was five, approximately one-fifth of what was obtained in the present work using TIS.

The use of liquid medium has been reported for other species of Lamiaceae family. For example, Grzegorczyk & Wysokiska [26] compared the induction and development of *Salvia officinalis* shoots in two liquid medium systems, with and without stirring, supplemented with 0.1 mg L^−1^ IAA and 0.45 mg L^−1^ 6-BAP. The results showed that in the shaken cultures, the necrosis and hyperhydricity of explants were higher. These effects could be caused by shear stress and being completely immersed in the liquid medium. In contrast, in static cultures, where the plants were partially submerged, there were no symptoms of hyperhydricity after three weeks.

Regarding the plant growth regulators used in this study, the optimum level for adventitious shoots formation of *R. officinalis* was 5 mg L^−1^ 6-BAP. This hormone is the most widely used and most effective cytokinin for the micropropagation of various plant species of economic interest, such as some Lamiaceae species [27]. For example, in the Basil (*Ocimum gratissimum* L.) carried out by Saha et al. [28], the best response was obtained with 1.0 mg L^−1^ 6-BAP (5.17 shoots per explant) after 12 days. The same effect was observed in *Thymus vulgaris* L. [29] and *Thymus mastichina* [30] in vitro cultures. However, in a study carried out with *Lavandula pudunculata* L., an excess of this hormone had an inhibitory effect on shoot elongation. For instance, the greatest number of shoots per explant was 4.07 using 0.5 mg L^−1^ of 6-BAP, but they were smaller than those obtained with 0.25 mg L^−1^ [31].

According to previous published results, it was decided to compare the phytohormones 6-BAP and NAA in the present work.

### 2.2. Evaluation of Different Immersion Frequencies

Once it was established the best in vitro proliferation conditions for *R. officinalis* shoots, the effect of different immersion periods (1, 5 and 10 min) and frequency (every 12 and 24 h) in liquid media supplemented with 5 mg L^−1^ of 6-BAP was evaluated.

After 72 days of culture, the best results were obtained with an immersion cycle of 1 min every 12 h, yielding 170.33 shoots (Table 2, entry 1). Fewer shoots were observed using the 24-h immersion frequency (Table 2, entries 2 and 4) compared to 12 h (Table 2, entries 1 and 3). No significant differences were observed with longer immersion times (Table 2, entries 5 and 6). It is possible that by increasing the duration of each immersion, oxygen deficiencies were originated in the plant tissues, causing a decrease in the shoots induction (Table 2, entries 1, 3 and 5). On the contrary, when the immersion times are short, the explants are covered with a film of liquid medium, which prevents their desiccation, reducing the resistance to diffusion of gases and continuously exchanging the gases between the plant tissue and the environment [32,33]. It is essential to note that although we observed a considerable increase from day 18 in the number, length and vigour of shoots produced in the culture, we decided to continue the experiment until 70 days because we didn’t observe necrosis or hyperhydricity on the explants.

The cycles, frequencies, and duration of the immersions influence the success of micropropagation in liquid medium because they favour the consumption of nutrients and water and reduce the hyperhydricity of the cultivated material. In cases where this cellular alteration has been observed, it can be eliminated or controlled in temporary immersion systems by adjusting the immersion times [34,35]. In addition, the movement of explants in the TIS during the immersions reduces the apical dominance and favours the separation of the tissues, which improves the generation of shoots. In general, the immersion cycles depend on various factors such as the plant species, the micropropagation process, and the TIS used. In *Musa* spp. using a TIS, no significant differences were observed in shoot production per explant with 4 min immersions every 3, 5, and 7 h, but in the latter treatment, shoots were longer [36]. In *Capsicum chinense*, the multiplication rate was increased using a temporary immersion bioreactor with 2 min immersions every eight hours [37].

Our results showed that *R. officinalis* responds best to shorter times (1 min) and more frequent immersions (every 12 h). This outcome may prevent hyperhydricity and result in the production of vigorous plants even after 72 days of the experiment.

### 2.3. Quantification of Carnosol in Callus Extracts Obtained by Maceration

Carnosol was identified by comparison of retention times. For quantitative analysis and response factor calculation, the standard addition method was used with carnosol as analyte. In preliminary experiments, we tried several mobile phases compositions to optimize carnosol detection in a complex mixture. Finally, eluate conditions were a flow rate of 1.2 mL min^−1^ using binary isocratic mobile phase system 60:40 (*v*/*v*) 10 mM formic acid (pH 3.0) (A) and acetonitrile (B) UV spectra were collected, and the quantification wavelength of these chromatograms was set at 254 and 293 nm. The injection volume was 20 µL. The selected isocratic chromatographic conditions allowed a fast carnosol determination with good resolution (Figure S7).

Once the best chromatographic conditions were established, the method was validated following the requirements of the International Conference on Harmonization (ICH) recommendations [34]. The analytical characteristics of the method presented in Table 3 are in accordance with the criteria established by the ICH [38].

The selectivity was evaluated by comparing the carnosol standard and a *R. officinalis* acetonic extracts samples chromatograms (Figure S8). The evaluation of these chromatograms showed that the carnosol peak did not interfere with other analytes present in the *R. officinalis* sample.

To determine the linear range in the present study, six different concentrations of carnosol (between 150 to 385 μg mL^−1^) were used to generate a calibration curve. Each solution was injected by triplicate and the linear coefficients, evaluated by the least square regression coefficients (R^2^), was 0.9994, evidencing the linearity of the method in this range.

Limits of quantification (LOQ) and detection (LOD) were experimentally calculated according to the standard deviation of the intercept with the y-axis (0.0256) and the slope (0.005) of the calibration curve. The LOD and LOQ were 3.78 and 12.62 μg m L^−1^, respectively (Table S1). The LOQ was smaller than the first point of the calibration curve (≤150 μg mL^−1^), confirming that both accurate and precision values throughout the working range were acceptable [38].

To evaluate the precision of the analytical method, the intra-day and the inter-day repeatability were evaluated as the relative standard deviation (RSD %) (Table 4). The RSD values varied from 0.73–1.37 % and 0.78–1.54% for both inter-day and intra-day precisions, respectively. Al these data evidenced an acceptable precision of the method for all concentrations assayed for both intraday and interday samples.

After preliminary thin layer chromatography (TLC) analysis of the 12 fractions obtained during the evaluation of the different culture systems (Table 1), we decided to analyze by HPLC the explants obtained from a liquid medium supplemented with 6-BAP at 2.5 (Table 1, entry 4) and 5.0 mg L^−1^ (Table 1, entry 6) (Figure S3). In the case of treatments where NAA were used, the concentration of carnosol by TLC was insignificant. The carnosol concentration was determined according to the developed method in *R. officinalis* wild and tissue culture samples. Carnosol contents in those samples were determined by the standard addition method and summarized in Table 5.

As shown in Table 5, although no significant differences were observed in both treatments, there was substantial production of carnosol in shoot culture (Table 5, entries 1 and 4) compared to the wild type plant extract (Table 3, entry 7). In the in vitro cultures, the content of carnosol varied from 0.7574 to 0.7771 mg g^−1^ biomass dry weight, and it was around 8-fold higher than in the wild plant. This increase could be originated due to in vitro growth conditions such as the composition of the culture medium, environmental conditions and response to stress subjected under this environment [39].

According to Grzegorczyk et al., the concentration of carnosol is closely related to shoot differentiation in *S. officinalis*. The highest diterpenoid yield (1.1 mg g^−1^ dry weight) was obtained in shoots of 10-week-old micropropagated plants. In our study, the carnosol concentration is 1.4 times lower, although our explants were only 7-weeks-old [40].

Santos et al. [41] discovered that in *Salvia officinalis* shoots obtained by in vitro culture techniques from six-week-old seedlings, carnosic acid and carnosol concentration depended on the amount of cytokinin supplemented to the culture medium. Moreover, the results obtained by Kuhlman and Röhl [42] indicate a higher concentration of carnosic acid and carnosol in rosemary shoots than in sage shoots (ranges from 5 to 42 times more). However, the concentration of carnosic acid was ten times lower than that of carnosol. On the other hand, Santos et al. [43] observed that after six months of subculturing sage calluses, carnosic acid and carnosol accumulation were 50 to 120 times higher than the levels found in rosemary calluses. In cell cultures suspensions, only both compounds were determined at trace levels. From these results, it is established that the synthesis of carnosol and carnosic acid depends on the level of cell differentiation in the in vitro culture.

## 3. Materials and Methods

### 3.1. General Experimental Procedures

Analytical thin-layer chromatography (TLC) was carried out on precoated silica gel 60F_254_ aluminium base plates (20 × 20 cm) (Merck, Kenilworth, NJ, USA). All solvents and reagents used in this work were purchased from Sigma-Aldrich Co (St. Louis, MO, USA). and used without further purification. Carnosol standard was procured from Sigma-Aldrich (St. Louis, MO, USA).

### 3.2. Material and Culture

Adult *R. officinalis* plants were used as the source of internodal segments, washed in running tap water for 3 min and then immersed in 70% (*v*/*v*) EtOH for 30 s, followed by Clorox (1.6 % NaClO) for 5 min. After sterilizing, the explants were rinsed in sterilized distilled water (twelve times in an hour) to remove all traces of the disinfectants. The disinfected explants were aseptically cultured on an MS Basal Medium to which sucrose (30 g L^−1^) and agar (only for solid medium experiments, 8 g L^−1^) were added [44]. The MS medium was supplemented with 6-benzylaminopurine (6-BAP) or α-naphthaleneacetic acid (NAA) in different concentrations for callus induction.

### 3.3. Experimental Design

To examine how the culture medium (solid and liquid) and different levels of 6-BAP (0.2, 2.5 and 5.0 mg/L) and NAA (0.5, 1.0 and 2.5 mg/L) affects the multiplication of *R. officinalis*, we designed an experiment of twelve treatments with three replicates each (Table 1). The cultures were incubated at 27 °C in dark and light (photoperiods of 16 h light). In the cultures with a liquid medium, an immersion of 1 min was made every 24 h. For the TIS, we evaluated the frequency (every 12 and 24 h) and the immersion time (1, 5 and 10 min) (Table 2). After 30 days of culture, the number of shoots per explant was counted for each treatment for all treatments.

### 3.4. Statistical Analysis

For all trials, the experimental design was completely randomized with three replicates. The data for the number of shoots obtained was subjected to analysis of variance (ANOVA) using the statistical program SPSS (Version 11.5 for Windows Inc., Chicago, IL, USA). To detect differences among treatments, Tukey’s test was used (*p* < 0.05).

### 3.5. Extraction of Carnosol and TLC Analysis

Aerial parts of wild and the in vitro explants were dried in the shade to a constant weight (one week, r.t.), then were ground and shaked with distilled acetone (10 g/100 mL, three times) at 250 rpm for 8 h at room temperature, following a method reported in the literature [45]. Combined filtrates were then removed under reduced pressure at 40 °C. All samples were stored at 4 °C until analysis.

Analytical thin-layer chromatography (TLC). The spotted samples were developed in a chromatography chamber saturated with mobile phase dichloromethane: acetone (95:5). The obtained chromatogram was then analyzed, and the R_f_ of the spots noted. Different dilute solutions were prepared using the stock solution of carnosol and developed in the saturated chamber as references. The fluorescence of the spots was recorded at 254 nm. The TLC was revealed with oleum (sulfuric acid, water, and acetic acid in the ratio 1:4:20 [*v*:*v*:*v*]) and heated at approximately 150 °C.

### 3.6. Instrumentation and HPLC Conditions

An HPLC instrument consisted of a BAS liquid chromatography system [Bioanalytical Systems Inc. (BAS), West Lafayette, IN, USA] consisting of a CC-5 liquid chromatography module, a PM 80 solvent delivery system, a diode array detector and Chromgraph 1.0.01 software (Liquid Chromatography Control Software) from BAS (West Lafayette, IN, USA) was used to record data. The system was equipped with a Hypersil C18 BDS column (250 × 4.6 mm, 5 μm; Thermo Scientific, Milford, MA, USA). The mobile phase was 10 mM formic (pH 3) (A) and acetonitrile (B) with an isocratic elution of 60:40. The chromatography was performed at room temperature (25 °C) for 30 min at a flow rate of 1.2 mL/min. The injection volume was 20 μL, and the wavelength of the detector was set at 254 and 293 nm. Stock solutions were prepared in acetonitrile. All solutions were filtered prior to analysis through a 0.2 µm syringe filter (Whatman) and injected three times into the HPLC column.

### 3.7. Preparation of Standards and Samples

The carnosol stock solution was prepared in methanol (733 µg mL^−1^) and diluted to obtain a range of concentrations from 0.150 to 0.385 µg mL^−1^. Rosemary extracts were dissolved in methanol (1 mL). All solutions were filtered through a 0.45 μm nylon membrane and stored at 4 °C before analysis.

### 3.8. Validation of HPLC Method

The HPLC method was validated following the ICH recommendations [38], with emphasis on specificity, linearity, trueness, precision, limit of detection (LOD) and quantification (LOQ). The specificity was assessed by comparing the chromatograms obtained for the acetonic extracts of R. officinalis samples with chromatograms of a carnosol solution to confirm that no ingredients could interfere with the target analyte. Linearity of the method was determinated from the construction of six points cali-bration curves in the range of 150 to 385 μg mL^−1^ (performed in triplicate). Six dilutions of carnosol standard in the range of 150 to 385 μg mL^−1^ were analyzed (performed in triplicate). The absorbance values of each concentration were measured at the selected wavelengths. The absorbance values obtained were plotted against the corresponding concentrations of carnosol, and they were evaluated by linear regression analysis based on the least-squares method (R^2^). The compound identification was based on the retention time. Limits of Detection (LOD) and Quantification (LOQ) were calculated based on both the residual standard deviation (σ) and the slope of the curve (s) from the linearity studies calibration curve according to equations: LOD = 3.3 σ/s and LOQ = 10 σ/s. To evaluate the precision of the method, intra-day and inter-day repeatability of retention time and concentration of carnosol were determined. Each solution of carnosol (50, 100 and 190 μg mL^−1^) were injected at six consecutive times of the same day and at six successive days under selected conditions. The precision was expressed as mean ± standard deviation (SD) and relative standard deviation (RSD).

### 3.9. Quantification of Carnosol

The H-Point Standard Addition Method (HPSAM) and linear regression was applied for the determination of carnosol [46]. Crude extract samples were prepared and transferred into three separate 1 mL volumetric flasks, to which was added a different volume of the standard solution (0, 20.0 and 40.0 µL). The absorbance of each solution was measured at the selected wavelength pair. The concentration of carnosol in the samples were calculated from the straight-line equation obtained from added concentration versus the ΔA values.

## 4. Conclusions

The results indicate that the micropropagation of rosemary in TIS has a better yield in the number of shoots compared to the solid medium, inducing 170.33 and 5.67 shoots per explant, respectively. TIS produced the maximum number of shoots per explant on a liquid medium supplemented with 6-BAP at 5.0 mg L^−1^, with an immersion cycle of 1 min every 12 h.

The HPLC method used allowed the separation and identification of carnosol in the rosemary extracts with a retention time of the carnosol of 10.95 min, observing an adequate separation and definition of the analyte signal.

It’s important to indicate that the content of carnosol in the in vitro cultures was around 8-fold higher than in the wild plant. The results showed that the in vitro culture of rosemary allows to obtain large amounts of buds and carnosol, which represents an efficient tool in the production and biosynthesis of natural antioxidants of added value. The present study represents an efficient alternative method to obtain carnosol for its pre-clinical and clinical development as a new antimicrobial agent.

## Figures and Tables

**Table 1:** Culture system and plant growth regulator effects on shoot number in *R. officinalis*.

Entry	Culture Medium	PGR ^1^ (mg L^−1^)	No. of Shoots ^2^
1	Solid	0.2/6-BAP	5.67 ± 0.58
2	TIS ^3^	0.2/6-BAP	6.67 ± 1.15
3	Solid	2.5/6-BAP	5.00 ± 1.00
4	TIS	2.5/6-BAP	15.00 ± 1.73
5	Solid	5.0/6-BAP	3.33 ± 1.15
6	TIS	5.0/6-BAP	24.33 ± 1.15
7	Solid	0.5/NAA	3.45 ± 1.27
8	TIS	0.5/NAA	5.67 ± 0.58
9	Solid	1.0/NAA	1.33 ± 1.15
10	TIS	1.0/NAA	9.08 ± 1.07
11	Solid	2.5/NAA	4.67 ± 2.31
12	TIS	2.5/NAA	6.67 ± 1.15

^1^ Plant growth regulator (PGR). ^2^ Average values ± SD. ^3^ The immersion time was programmed at 1 min with a frequency of every 24 h.

**Table 2:** Effects of immersion time and frequency on the number of shoots per explant of *R. officinalis*.

Entry	Immersion Frequencies	Immersion Time (min)	No. of Shoots ^1^
1	every 12 h	1	170.33 ± 29.40
2	every 24 h	1	3.33 ± 1.15
3	every 12 h	5	22.00 ± 30.41
4	every 24 h	5	5.33 ± 3.06
5	every 12 h	10	5.67 ± 0.58
6	every 24 h	10	7.00 ± 7.94

^1^ Average values ± SD.

**Table 3:** System suitability of the HPLC method (*n* = 6).

Chromatographic Parameter	Result	Acceptance Criteria
Number of theorical plates (N)	2862.3 ± 18.9	N > 2000
Tailing factor (T)	1.07 ± 0.01	T ≤ 2
Retention factor (k)	3.01 ± 0.01	k > 2

**Table 4:** Precision parameters expressed as RSD % (*n* = 6).

Carnosol Sample Solution (µg/mL)	Quantified Concentration ± SD (µg/mL)	Recovery ± SD (%)	RSD (%)
Intra-day precision			
50	47.74 ± 0.51	95.5	1.07
100	97.9 ± 1.34	97.9	1.37
190	191.1 ± 1.40	100.6	0.73
	Inter-day precision		
50	48.65 ± 0.75	97.3	1.54
100	98.48 ± 0.94	98.5	0.95
190	190.11 ± 1.49	100.1	0.78

**Table 5:** Content of carnosol (CA) in the acetonic extracts of wild plant and callus samples of *R. officinalis*.

Entry	Treatment	Retention Time (min)	Retention Factor	Absorbance (AU)	Carnosol Concentration (μg mL^−1^) ^1^	Carnosol Concentration as mg g^−1^ DW ^2^
1	6-BAP (2.5 mgL^−1^)	9.16 ± 0.001	2.77	0.400 ± 0.001	77.71 ± 0.13	0.7771
2	6-BAP (2.5 mgL^−1^) + 28.87 μg mL^−1^ CA	9.13 ± 0.004	2.76	0.527 ± 0.006	101.56 ± 1.24	-
3	6-BAP (2.5 mgL^−1^) + 53.33 μg mL^−1^ CA	9.15 ± 0.002	2.77	0.650 ± 0.001	126.27 ± 0.30	-
4	6-BAP (5.0 mgL^−1^)	8.97 ± 0.023	2.69	0.387 ± 0.001	75.74 ± 0.33	0.7574
5	6-BAP (5.0 mgL^−1^) + 28.87 μg mL^−1^ CA	9.01 ± 0.009	2.71	0.463 ± 0.004	90.61 ± 0.78	-
6	6-BAP (5.0 mgL^−1^) + 53.33 μg mL^−1^ CA	9.00 ± 0.012	2.70	0.551 ± 0.001	107.1 ± 0.75	-
7	Wild plant	9.11 ± 0.004	2.75	0.050 ± 0.001	9.68 ± 0.21	0.0968
8	CA (173.24 μg mL^−1^)	9.21 ± 0.011	2.79	0.892 ± 0.001	173.24 ± 0.39	

^1^ Average values ± SD. ^2^ The concentration of carnosol expressed as milligrams of carnosol per gram of plant dry weight (mg/g DW).

## Data Availability

Authors can confirm that all relevant data are included in the article.

## Supplementary Materials

The following are available online at https://www.mdpi.com/article/10.3390/ph14080747/s1, Figure S1: Effects of 5 mg L^−1^ 6-BAP on induction of shoots from explants of *R. officinalis* L, Figure S2: Effects of 5 mg L^−1^ 6-BAP with an immersion cycle of 1 min every 12 h on induction of shoots from explants of *R. officinalis* L., Table S1: Solid culture system and plant growth regulator effects on shoot number in *R. officinalis*, Table S2: Liquid culture system and plant growth regulator effects on shoot number in *R. officinalis*, Table S3: Effects of immersion time and frequency on the contamination, necrosis, number of callus and shoots, Figure S3: Effect of 6-BAP and NAA on callus induction in *R. officinalis* explants versus days of cultivation, Figure S4: Effect of 6-BAP and NAA on number of shoots in *R. officinalis* explants versus days of cultivation, Figure S5: Thin-layer chromatography of acetonic extracts of callus samples from fractions 1–12, Table S4: Validation parameters for the determination of carnosol by HPLC-DAD, Table S5: Influence of the flow of the mobile on retention time (tR), dead time (t0) and retention factors (k’), Figure S6: Calibration curve for carnosol, Figure S7: Chromatogram of carnosol at 370 μg mL^−1^, Figure S8: Chromatogram of the Carnosol standard at 220 mg mL^−1^ vs. chromatogram of a sample of *R. officinalis* acetonic extract, Figure S9: Characteristic HPLC profile of the acetonic extract of a callus sample of *R. officinalis*.
