# Supplementary material for: In Vitro Culture of Rosmarinus officinalis L. in a Temporary Immersion System: Influence of Two Phytohormones on Plant Growth and Carnosol Production
Source: Pharmaceuticals (Basel). 2021 Jul 29;14(8):747. doi: 10.3390/ph14080747 (PMC8398425; doi:10.3390/ph14080747)
Supplement: Supplementary file 1 [file pharmaceuticals-14-00747-s001.zip › pharmaceuticals-1239894-supplementary.pdf]

In vitro Culture of *Rosmarinus Officinalis* L. in a Temporary Immersion System: Influence of two Phytohormones on Plant Growth and Carnosol Production

Eder Villegas Sánchez, Mariana Macías-Alonso, Soraya Osegueda Robles, Lisset Herrera-Isidrón, Hector Nuñez-Palenius & Joaquín G. Marrero.

Supplementary information

Table of contents

- General Experimental Procedures S2
- Plant Material S2
- Figure S1. S2
- Figure S2. S2
- Table S1. S3
- Table S2. S3
- Table S3. S4
- Figure S3. S5
- Figure S4. S5
- Figure S5. S6
- Table S4. S6
- Table S5. S7
- Figure S6. S7
- Figure S6. S8

General Experimental Procedures

All solvents and reagents used in this work were purchased from Sigma-Aldrich Co. and used without further purification. Carnosol standard was procured from Sigma-Aldrich. Analytical thin-layer chromatography (TLC) were carried out on precoated silica gel 60F254 aluminium base plates with a 230-400 mesh particle size (20 x 20 cm) (Merck), using a combination of dichloromethane (Cl_2CH_2) and acetone (Me_2CO) as an eluent, in proportion 95:5. In all cases, the TLC plates were visualized by exposure to ultra violet light, and then the spots were revealed by spraying with oleum (80% acetic acid, 16% water, 4% sulphuric acid) and heating at 120 °C for 15 minutes.

Plant Material

Wild plants were collected in the ethnobotanical garden Francisco Peláez Roldán, located at street 2 South 1700 in the city of San Andrés Cholula, Puebla, and a specimen of *Rosmarinus officinalis* was deposited in the same ethnobotanical garden.

Shoots regenerated from nodal explants

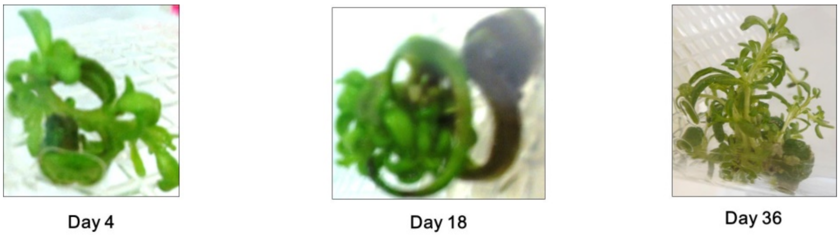

Figure S1. Effects of 5 mg L^{-1} 6-BAP on induction of shoots from explants of *R. officinalis* L.

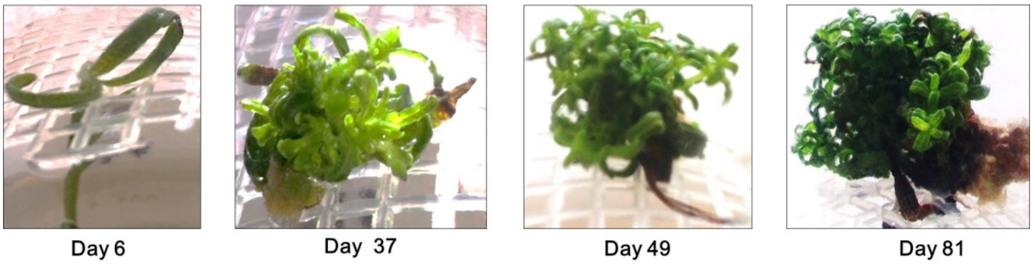

Figure S2. Effects of 5 mg L^{-1} 6-BAP with an immersion cycle of 1 min every 12 h on induction of shoots from explants of *R. officinalis* L.

Table S1. Solid culture system and plant growth regulator effects on shoot number in *R. officinalis*.

Plant growth regulator		6-BAP														
Concentration (mg/L)		0.2					2.5					5				
	Day	1A	1B	1C	PROM.	Desv Std	2A	2B	2C	PROM.	Desv Std	3A	3B	3C	PROM.	Desv Std
% Contamination	0	0	0	0	0.00	0.00	0	0	0	0.00	0.00	0	0	0	0.00	0.00
	4	0	0	0	0.00	0.00	0	0	0	0.00	0.00	0	0	0	0.00	0.00
	7	0	0	0	0.00	0.00	0	0	0	0.00	0.00	0	0	0	0.00	0.00
	10	0	0	0	0.00	0.00	0	0	0	0.00	0.00	0	0	0	0.00	0.00
	17	0	0	0	0.00	0.00	0	0	0	0.00	0.00	0	0	0	0.00	0.00
	28	0	0	0	0.00	0.00	0	0	0	0.00	0.00	0	0	0	0.00	0.00
% Necrosis	0	0	0	0	0.00	0.00	0	0	0	0.00	0.00	0	0	0	0.00	0.00
	4	15	1	5	7.00	7.21	40	1	15	18.67	19.76	0	40	60	33.33	30.55
	7	20	1	10	10.33	9.50	45	5	15	21.67	20.82	0	45	60	35.00	31.22
	10	20	1	10	10.33	9.50	55	10	35	33.33	22.55	0	75	65	46.67	40.72
	17	28	9	17	18.00	9.54	70	30	45	48.33	20.21	100	100	50	83.33	28.87
	28	30	24	17	23.67	6.51	98	42	48	62.67	30.75	100	100	100	100.00	0.00
No. Callus	0	2	4	1	2.33	1.53	0	0	0	0.00	0.00	0	0	0	0.00	0.00
	4	2	4	1	2.33	1.53	0	0	0	0.00	0.00	0	0	0	0.00	0.00
	7	2	4	1	2.33	1.53	0	0	0	0.00	0.00	0	0	0	0.00	0.00
	10	2	4	1	2.33	1.53	0	0	0	0.00	0.00	0	0	0	0.00	0.00
	17	2	4	2	2.67	1.15	0	0	0	0.00	0.00	1	1	2	1.33	0.58
	28	2	4	2	2.67	1.15	0	0	0	0.00	0.00	1	1	4	2.00	1.73
No. Shoots	0	2	5	6	4.33	2.08	4	4	2	3.33	1.15	2	2	2	2.00	0.00
	4	2	5	6	4.33	2.08	4	4	2	3.33	1.15	4	2	2	2.67	1.15
	7	2	5	6	4.33	2.08	4	6	4	4.67	1.15	4	2	2	2.67	1.15
	10	3	5	6	4.67	1.53	4	6	4	4.67	1.15	4	2	2	2.67	1.15
	17	4	5	6	5.00	1.00	4	6	4	4.67	1.15	5	2	2	3.00	1.73
	28	6	5	6	5.67	0.58	5	6	4	5.00	1.00	5	2	2	3.00	1.73

Plant growth regulator		NAA														
Concentration (mg/L)		0.5					1					2.5				
	Day	4A	4B	4C	PROM.	Desv Std	5A	5B	5C	PROM.	Desv Std	6A	6B	6C	PROM.	Desv Std
% Contamination	0	0	0	0	0.00	0.00	0	0	0	0.00	0.00	0	0	0	0.00	0.00
	4	0	0	0	0.00	0.00	0	0	0	0.00	0.00	0	0	0	0.00	0.00
	7	0	0	0	0.00	0.00	0	0	0	0.00	0.00	0	0	0	0.00	0.00
	10	0	0	0	0.00	0.00	0	0	0	0.00	0.00	0	0	0	0.00	0.00
	17	0	0	0	0.00	0.00	0	0	0	0.00	0.00	0	0	0	0.00	0.00
	28	0	0	0	0.00	0.00	0	0	0	0.00	0.00	0	0	0	0.00	0.00
% Necrosis	0	0	0	0	0.00	0.00	0	0	0	0.00	0.00	0	0	0	0.00	0.00
	4	5	15	15	11.67	5.77	10	0	0	3.33	5.77	40	30	5	25.00	18.03
	7	30	15	15	20.00	8.66	20	0	10	10.00	10.00	40	40	15	31.67	14.43
	10	50	60	20	43.33	20.82	30	0	20	16.67	15.28	60	40	40	46.67	11.55
	17	85	100	30	71.67	36.86	85	0	55	46.67	43.11	100	65	90	85.00	18.03
	28	100	100	100	100.00	0.00	100	100	100	100.00	0.00	100	100	100	100.00	0.00
No. Callus	0	0	0	0	0.00	0.00	0	1	1	0.67	0.58	0	0	0	0.00	0.00
	4	0	0	0	0.00	0.00	0	1	1	0.67	0.58	0	0	0	0.00	0.00
	7	0	0	0	0.00	0.00	0	1	1	0.67	0.58	0	0	0	0.00	0.00
	10	0	0	0	0.00	0.00	0	1	1	0.67	0.58	0	0	0	0.00	0.00
	17	3	0	2	1.67	1.53	2	2	1	1.67	0.58	1	2	5	2.67	2.08
	28	3	0	3	2.00	1.73	3	3	3	3.00	0.00	1	4	11	5.33	5.13
No. Shoots	0	2	2	2	2.00	0.00	2	0	2	1.33	1.15	6	6	2	4.67	2.31
	4	2	3	2	2.33	0.58	2	0	2	1.33	1.15	6	6	2	4.67	2.31
	7	2	3	2	2.33	0.58	2	0	2	1.33	1.15	6	6	2	4.67	2.31
	10	2	4	2	2.67	1.15	2	0	2	1.33	1.15	6	6	2	4.67	2.31
	17	2	4	2	2.67	1.15	2	0	2	1.33	1.15	6	6	2	4.67	2.31
	28	2	4	4	3.33	1.15	2	0	2	1.33	1.15	6	6	2	4.67	2.31

Table S2. Liquid culture system and plant growth regulator effects on shoot number in *R. officinalis*.

Plant growth regulator		6-BAP																				
Concentration (mg/L)		0.2							2.5							5						
	Day	1A	1B	1C	1E	1D	PROM.	Desv Std	2B	2A	2C	2D	2E	PROM.	Desv Std	3A	3B	3C	3E	3D	PROM.	Desv Std
% Contamination	0	0	0	0	0	0	0.00	0.00	0	0	0	0	0	0.00	0.00	0	0	0	0	0	0.00	0.00
	4	0	0	0	0	0	0.00	0.00	0	0	0	0	0	0.00	0.00	0	0	0	0	0	0.00	0.00
	7	0	0	0	0	0	0.00	0.00	0	0	0	0	0	0.00	0.00	0	0	0	0	0	0.00	0.00
	10	100	0	0	0	0	0.00	0.00	0	0	0	0	0	0.00	0.00	0	0	0	0	0	0.00	0.00
	17	100	0	0	0	0	0.00	0.00	0	0	0	0	0	0.00	0.00	0	0	0	0	0	0.00	0.00
	24	100	0	0	0	0	0.00	0.00	0	0	0	0	0	0.00	0.00	0	0	0	0	0	0.00	0.00
	28	100	0	0	0	0	0.00	0.00	0	0	0	0	0	0.00	0.00	0	0	0	0	0	0.00	0.00
	42	100	0	0	0	0	0.00	0.00	0	0	0	0	0	0.00	0.00	0	0	0	0	0	0.00	0.00
% Necrosis	0	0	0	0	0	0	0.00	0.00	0	0	0	0	0	0.00	0.00	0	0	0	0	0	0.00	0.00
	4	0	1	5	25	1	10.33	12.86	1	5	0	5	0	3.33	2.89	1	10	5	1	5	5.33	4.51
	7	1	1	10	30	1	13.67	14.84	1	5	5	5	5	5.00	0.00	1	15	5	1	5	7.00	7.21
	10	1	5	25	100	1	43.33	50.08	5	10	10	5	5	8.33	2.89	5	15	5	1	5	8.33	5.77
	17	---	10	35	100	10	48.33	46.46	5	10	10	5	5	8.33	2.89	5	15	5	1	5	8.33	5.77
	24	---	75	95	100	75	90.00	13.23	45	60	25	10	5	31.67	25.66	50	40	20	5	10	36.67	15.28
	28	---	95	95	100	95	96.67	2.89	45	70	50	40	15	53.33	15.28	55	45	25	5	10	41.67	15.28
	42	---	100	100	100	100	100.00	0.00	65	90	70	50	15	70.00	20.00	90	65	50	5	10	68.33	20.21
No. Callus	0	0	0	1	0	1	0.33	0.58	0	0	2	0	1	0.67	1.15	2	0	1	1	2	1.00	1.00
	4	0	0	1	0	1	0.33	0.58	0	0	1	0	1	0.33	0.58	2	0	1	1	2	1.00	1.00
	7	0	1	1	0	1	0.67	0.58	3	1	2	1	2	1.33	0.58	2	0	1	1	2	1.00	1.00
	10	0	1	1	0	1	0.67	0.58	3	1	2	2	2	1.67	0.58	3	1	1	2	2	1.67	1.15
	17	---	1	1	0	1	0.67	0.58	3	1	2	3	2	2.00	1.00	3	2	2	2	2	2.33	0.58
	24	---	1	1	0	1	0.67	0.58	3	1	5	5	2	3.67	2.31	3	2	2	2	2	2.33	0.58
	28	---	1	1	0	1	0.67	0.58	3	1	5	5	2	3.67	2.31	3	2	2	2	2	2.33	0.58
	42	---	1	1	0	1	0.67	0.58	3	1	5	5	2	3.67	2.31	3	2	2	2	2	2.33	0.58
No. Shoots	0	3	2	4	2	6	2.67	1.15	6	2	6	6	6	4.67	2.31	2	6	2	3	2	3.33	2.31
	4	5	4	4	4	8	4.00	0.00	7	5	6	7	11	6.00	1.00	4	6	2	5	4	4.00	2.00
	7	6	4	6	6	10	5.33	1.15	8	8	6	10	15	8.00	2.00	6	6	6	5	6	6.00	0.00
	10	6	4	6	6	10	5.33	1.15	11	8	8	14	15	10.00	3.46	6	6	6	7	6	6.00	0.00
	17	---	6	6	6	13	6.00	0.00	14	9	8	14	17	10.33	3.21	8	14	8	14	15	10.00	3.46
	24	---	6	6	6	13	6.00	0.00	22	13	14	17	18	14.67	2.08	14	16	14	18	19	14.67	1.15
	28	---	8	6	6	13	6.67	1.15	22	14	14	17	23	15.00	1.73	15	19	15	21	29	16.33	2.31
	42	---	8	6	6	13	6.67	1.15	22	14	14	17	29	15.00	1.73	25	23	25	30	39	24.33	1.15

Plant growth regulator		NAA																				
Concentration (mg/L)		0.5							1							2.5						
	Day	4A	4B	4C	4D	4E	PROM.	Desv Std	5A	5B	5C	5D	5E	PROM.	Desv Std	6A	6B	6C	6D	6E	PROM.	Desv Std
% Contamination	0	0	0	0	0	0	0.00	0.00	0	0	0	0	0	0.00	0.00	0	0	0	0	0	0.00	0.00
	4	0	0	0	0	0	0.00	0.00	0	0	0	0	0	0.00	0.00	0	0	0	0	0	0.00	0.00
	7	0	0	0	0	0	0.00	0.00	0	0	0	0	0	0.00	0.00	0	0	0	0	0	0.00	0.00
	10	0	0	0	0	0	0.00	0.00	0	0	0	0	0	0.00	0.00	0	0	0	0	100	0.00	0.00
	17	0	0	0	0	0	0.00	0.00	0	0	0	0	0	0.00	0.00	0	0	0	0	100	0.00	0.00
	24	0	0	0	0	0	0.00	0.00	0	0	0	0	0	0.00	0.00	0	0	0	0	100	0.00	0.00
	28	0	0	0	0	0	0.00	0.00	0	0	0	0	0	0.00	0.00	0	0	0	0	100	0.00	0.00
	42	0	0	0	0	0	0.00	0.00	0	0	0	0	0	0.00	0.00	0	0	0	0	100	0.00	0.00
% Necrosis	0	0	0	0	0	0	0.00	0.00	0	0	0	0	0	0.00	0.00	0	0	0	0	0	0.00	0.00
	4	15	95	5	10	5	36.67	50.58	1	1	0	1	10	0.67	0.58	1	0	1	1	5	0.67	0.58
	7	20	100	15	10	10	41.67	50.58	1	1	1	1	15	1.00	0.00	5	5	1	1	10	2.33	2.31
	10	20	100	15	10	10	41.67	50.58	1	1	1	1	15	1.00	0.00	10	10	1	1	15	4.00	5.20
	17	25	100	25	15	15	46.67	46.46	1	1	1	5	25	2.33	2.31	10	10	1	10	---	7.00	5.20
	24	50	100	70	50	60	73.33	25.17	5	65	20	40	75	41.67	22.55	30	75	20	30	---	41.67	29.30
	28	65	100	75	50	100	75.00	25.00	10	65	40	45	80	50.00	13.23	35	95	90	50	---	78.33	24.66
	42	100	100	100	100	100	100.00	0.00	15	90	75	80	100	81.67	7.64	70	100	100	75	---	91.67	14.43
No. Callus	0	0	0	1	0	1	0.33	0.58	1	0	1	1	0	0.67	0.58	1	1	1	0	1	0.67	0.58
	4	0	0	1	0	1	0.33	0.58	1	1	1	3	0	1.67	1.15	1	1	1	0	1	0.67	0.58
	7	0	0	2	0	1	0.67	1.15	1	1	1	4	0	2.00	1.73	1	1	1	1	1	1.00	0.00
	10	0	0	2	1	1	1.00	1.00	1	1	1	4	0	2.00	1.73	1	1	2	1	2	1.33	0.58
	17	0	0	2	1	1	1.00	1.00	1	1	4	8	0	4.33	3.51	1	2	2	2	---	2.00	0.00
	24	1	0	2	1	1	1.00	1.00	2	1	5	9	0	5.00	4.00	4	2	2	2	---	2.00	0.00
	28	1	0	2	1	1	1.00	1.00	2	1	5	9	0	5.00	4.00	4	2	2	2	---	2.00	0.00
	42	1	0	2	1	1	1.00	1.00	2	1	5	9	0	5.00	4.00	4	2	2	2	---	2.00	0.00
No. Shoots	0	2	2	2	2	6	2.00	0.00	6	6	5	6	2	5.67	0.58	7	2	4	4	12	3.33	1.15
	4	3	4	4	3	8	3.67	0.58	10	9	5	10	3	8.00	2.65	9	3	6	4	12	4.33	1.53
	7	5	5	5	6	9	5.33	0.58	12	10	5	14	5	9.67	4.51	11	3	6	5	14	4.67	1.53
	10	5	5	6	6	9	5.67	0.58	13	10	8	14	5	10.67	3.06	13	4	6	5	14	5.00	1.00
	17	6	5	6	6	10	5.67	0.58	15	14	10	14	5	12.67	2.31	13	4	6	5	---	5.00	1.00
	24	7	5	6	6	10	5.67	0.58	17	16	10	14	6	13.33	3.06	13	6	6	8	---	6.67	1.15
	28	7	5	6	6	10	5.67	0.58	19	16	10	14	6	13.33	3.06	13	6	6	8	---	6.67	1.15
	42	7	5	6	6	10	5.67	0.58	22	16	15	14	6	15.00	1.00	13	6	6	8	---	6.67	1.15

Table S3. Effects of immersion time and frequency on the contamination, necrosis, number of Callus and shoots.

One Immersion every 24 HORAS — Plant growth regulators: 6-BAP; Concentration (mg/L): 2.5 mg/L

Immersion time (min)		1						5						10					
	Day	1A	1B	1D	1C	PROM.	Desv std	2B	2A	2C	2D	PROM.	Desv std	3A	3B	3C	3D	PROM.	Desv std
% Contamination	0	0	0	0	0	0.00	0.00	0	0	0	0	0.00	0.00	0	0	0	0	0.00	0.00
	5	0	0	0	0	0.00	0.00	0	0	0	0	0.00	0.00	100	0	0	0	0.00	0.00
	34	0	0	0	0	0.00	0.00	0	0	0	0	0.00	0.00	100	0	0	0	0.00	0.00
	48	0	0	0	0	0.00	0.00	0	0	0	0	0.00	0.00		0	0	0	0.00	0.00
	70	0	0	0	0	0.00	0.00	0	0	0	0	0.00	0.00		0	0	0	0.00	0.00
% Necrosis	0	0	1	2	1	1.00	1.00	0	0	0	8	2.50	4.33	0	0	0	0	0.00	0.00
	5	1	2	2	2	1.53	0.50	0	2	0	60	20.50	34.22	60	0	1	75	25.33	43.02
	34	2	3	5	100	3.33	1.53	4	15	100	90	68.33	46.46		3	100	100	67.67	56.00
	48	2	4	6	100	4.00	2.00	6	22	100	90	70.67	42.44		4	100	100	68.00	55.43
	70	7	6	6	100	6.33	0.58	6	54	100	100	84.67	26.56		17	100	100	72.33	47.92
No. of Callus	0	0	0	0	0	0.00	0.00	0	0	0	0	0.00	0.00	0	0	0	0	0.00	0.00
	5	1	0	0	0	0.33	0.58	1	0	0	0	0.00	0.00	0	0	0	0	0.00	0.00
	34	4	1	3	0	2.67	1.53	2	1	0	0	0.33	0.58		2	0	0	0.67	1.15
	48	4	2	3	0	3.00	1.00	4	2	0	0	0.67	1.15		2	0	0	0.67	1.15
	70	4	3	3	0	3.33	0.58	5	2	0	0	0.67	1.15		2	0	0	0.67	1.15
No. of Shoots	0	7	9	8	6	8.00	1.00	6	10	2	4	5.33	4.16	2	4	6	2	4.00	2.00
	5	7	12	8	6	9.00	2.65	6	10	4	8	7.33	3.06	2	4	12	2	6.00	5.29
	34	13	47	23	12	27.67	17.47	26	23	12	15	16.67	5.69		28	18	12	19.33	8.08
	48	26	73	86	12	61.67	31.56	67	33	19	26	26.00	7.00		43	23	25	30.33	11.02
	70	94	99	95	12	96.00	2.65	76	33	28	35	32.00	3.61		50	35	38	41.00	7.94

One Immersion every 24 HORAS — Plant growth regulators: 6-BAP; Concentration (mg/L): 5 mg/L

Immersion time (min)		1						5						10					
	Day	4B	4A	4C	4D	PROM.	Desv std	5B	5A	5C	5D	PROM.	Desv std	6A	6B	6C	6D	PROM.	Desv std
% Contamination	0	0	0	0	0	0.00	0.00	0	0	0	0	0.00	0.00	0	0	0	0	0.00	0.00
	5	100	0	0	0	0.00	0.00	0	0	0	0	0.00	0.00	0	0	0	0	0.00	0.00
	34	100	0	0	0	0.00	0.00	0	0	0	0	0.00	0.00	0	0	0	0	0.00	0.00
	48	100	0	0	0	0.00	0.00	0	0	0	0	0.00	0.00	0	0	0	0	0.00	0.00
	70	100	0	0	0	0.00	0.00	0	0	0	0	0.00	0.00	0	0	0	0	0.00	0.00
% Necrosis	0		28	3	28	19.67	14.43	0	0	27	6	11.00	14.18	0	20	10	0	10.00	10.00
	5		45	5	63	37.67	29.69	1	25	77	6	36.00	36.76	1	35	100	0	45.33	50.30
	34		88	100	100	96.00	6.93	11	100	95	66	87.00	18.36	15	100	100	2	71.67	49.07
	48		100	100	100	100.00	0.00	12	100	100	100	100.00	0.00	15	100	100	3	71.67	49.07
	70		100	100	100	100.00	0.00	88	100	100	100	100.00	0.00	100	100	100	6	100.00	0.00
No. of Callus	0	0	0	0	0	0.00	0.00	0	0	0	0	0.00	0.00	0	0	0	0	0.00	0.00
	5	0	0	0	0	0.00	0.00	1	0	0	0	0.00	0.00	1	0	0	0	0.33	0.58
	34		1	2	0	1.00	1.00	1	2	0	1	1.00	1.00	1	0	0	1	0.33	0.58
	48		1	2	0	1.00	1.00	1	2	0	1	1.00	1.00	1	0	0	1	0.33	0.58
	70		1	2	0	1.00	1.00	1	2	0	2	1.33	1.15	1	0	0	1	0.33	0.58
No. of Shoots	0	3	4	2	4	3.33	1.15	6	2	6	6	4.67	2.31	6	4	1	5	3.67	2.52
	5	3	4	2	4	3.33	1.15	12	2	6	6	4.67	2.31	6	4	1	6	3.67	2.52
	34		4	2	4	3.33	1.15	51	2	6	8	5.33	3.06	16	4	1	28	7.00	7.94
	48		4	2	4	3.33	1.15	60	2	6	8	5.33	3.06	16	4	1	52	7.00	7.94
	70		4	2	4	3.33	1.15	60	2	6	8	5.33	3.06	16	4	1	101	7.00	7.94

One Immersion every 12 HORAS — Plant growth regulators: 6-BAP; Concentration (mg/L): 2.5 mg/L

Immersion time (min)		1						5						10					
	Day	7A	7B	7C	7D	PROM.	Desv std	8A	8B	8C	8D	PROM.	Desv std	9A	9C	9B	9D	PROM.	Desv std
% Contamination	0	0	0	0	0	0.00	0.00	0	0	0	0	0.00	0.00	0	0	0	0	0.00	0.00
	5	0	0	0	0	0.00	0.00	0	0	0	0	0.00	0.00	0	0	0	0	0.00	0.00
	34	0	0	0	0	0.00	0.00	0	0	0	0	0.00	0.00	0	0	0	100	0.00	0.00
	48	0	0	0	0	0.00	0.00	0	0	0	0	0.00	0.00	0	0	0		0.00	0.00
	70	0	0	0	0	0.00	0.00	0	0	0	0	0.00	0.00	0	0	0		0.00	0.00
% Necrosis	0	2	0	4	0	2.00	2.00	50	20	4	8	24.67	23.35	13	0	20	0	11.00	10.15
	5	3	1	11	0	5.00	5.29	83	30	12	23	41.67	36.91	13	0	50	82	21.00	25.94
	34	6	40	77	5	41.00	35.51	100	100	60	98	86.67	23.09	36	5	50	82	30.33	23.03
	48	11	78	85	7	58.00	40.85	100	100	80	100	93.33	11.55	40	5	50		31.67	23.63
	70	12	100	85	7	65.67	47.08	100	100	80	100	93.33	11.55	100	5	50		51.67	47.52
No. of Callus	0	0	0	0	0	0.00	0.00	0	0	0	0	0.00	0.00	0	0	0	0	0.00	0.00
	5	0	0	0	0	0.00	0.00	0	0	0	0	0.00	0.00	0	0	0	0	0.00	0.00
	34	2	2	2	1	2.00	0.00	2	0	2	1	1.33	1.15	3	8	0	0	3.67	4.04
	48	2	2	2	1	2.00	0.00	2	0	2	1	1.33	1.15	3	8	0		3.67	4.04
	70	2	4	2	1	2.67	1.15	2	0	2	1	1.33	1.15	3	8	0		3.67	4.04
No. of Shoots	0	5	4	7	10	5.33	1.53	4	2	5	6	3.67	1.53	4	6	4	6	4.67	1.15
	5	5	4	7	10	5.33	1.53	4	2	5	6	3.67	1.53	4	6	4	6	4.67	1.15
	34	33	5	7	64	15.00	15.62	4	2	5	6	3.67	1.53	10	24	10	6	14.67	8.08
	48	33	5	8	139	15.33	15.37	4	2	5	6	3.67	1.53	12	54	12		26.00	24.25
	70	33	7	8	139	16.00	14.73	4	2	5	6	3.67	1.53	14	118	16		49.33	59.48

One Immersion every 12 HORAS — Plant growth regulators: 6-BAP; Concentration (mg/L): 5 mg/L

Immersion time (min)		1						5						10					
	Day	10C	10B	10D	10A	PROM.	Desv std	11B	11C	11A	11D	PROM.	Desv std	12C	12A	12B	12D	PROM.	Desv std
% Contamination	0	0	0	0	0	0.00	0.00	0	0	0	0	0.00	0.00	0	0	0	0	0.00	0.00
	5	0	0	0	0	0.00	0.00	100	0	0	0	0.00	0.00	100	0	0	0	0.00	0.00
	34	0	0	0	0	0.00	0.00	100	0	0	0	0.00	0.00		0	0	0	0.00	0.00
	48	0	0	0	0	0.00	0.00		0	0	0	0.00	0.00		0	0	0	0.00	0.00
	70	0	0	0	0	0.00	0.00		0	0	0	0.00	0.00		0	0	0	0.00	0.00
% Necrosis	0	8	0	0	0	0.00	0.00	10	20	0	0	6.67	11.55	0	56	7	0	21.00	30.51
	5	40	1	0	0	0.17	0.29	30	20	1	0	7.00	11.27	0	100	7	0	35.67	55.82
	34	100	7	1	1	3.00	3.46		93	4	43	46.67	44.61	9	100	83	100	94.33	9.81
	48	100	9	1	2	4.00	4.36		100	4	43	49.00	48.28	33	100	100	100	100.00	0.00
	70	100	10	2	2	4.67	4.62		100	4	43	49.00	48.28	100	100	100	100	100.00	0.00
No. of Callus	0	0	0	0	0	0.00	0.00	0	0	0	0	0.00	0.00	0	0	0	0	0.00	0.00
	5	0	0	0	0	0.00	0.00	1	1	0	1	0.67	0.58	1	0	0	1	0.33	0.58
	34	1	2	1	2	1.67	0.58		2	1	2	1.67	0.58	2	0	2	2	1.33	1.15
	48	2	5	2	5	4.00	1.73		3	1	3	2.33	1.15	2	0	2	2	1.33	1.15
	70	2	5	2	5	4.00	1.73		3	1	3	2.33	1.15	2	0	2	2	1.33	1.15
No. of Shoots	0	2	6	4	4	4.67	1.15	2	2	4	4	3.33	1.15	7	5	6	6	5.67	0.58
	5	2	6	4	4	4.67	1.15	2	2	4	4	3.33	1.15	7	5	6	6	5.67	0.58
	34	2	50	62	91	67.67	21.08		2	4	7	4.33	2.52	51	5	6	6	5.67	0.58
	48	2	97	148	143	129.33	28.11		2	12	7	7.00	5.00	71	5	6	6	5.67	0.58
	70	2	146	203	162	170.33	29.40		2	57	7	22.00	30.41	91	5	6	6	5.67	0.58

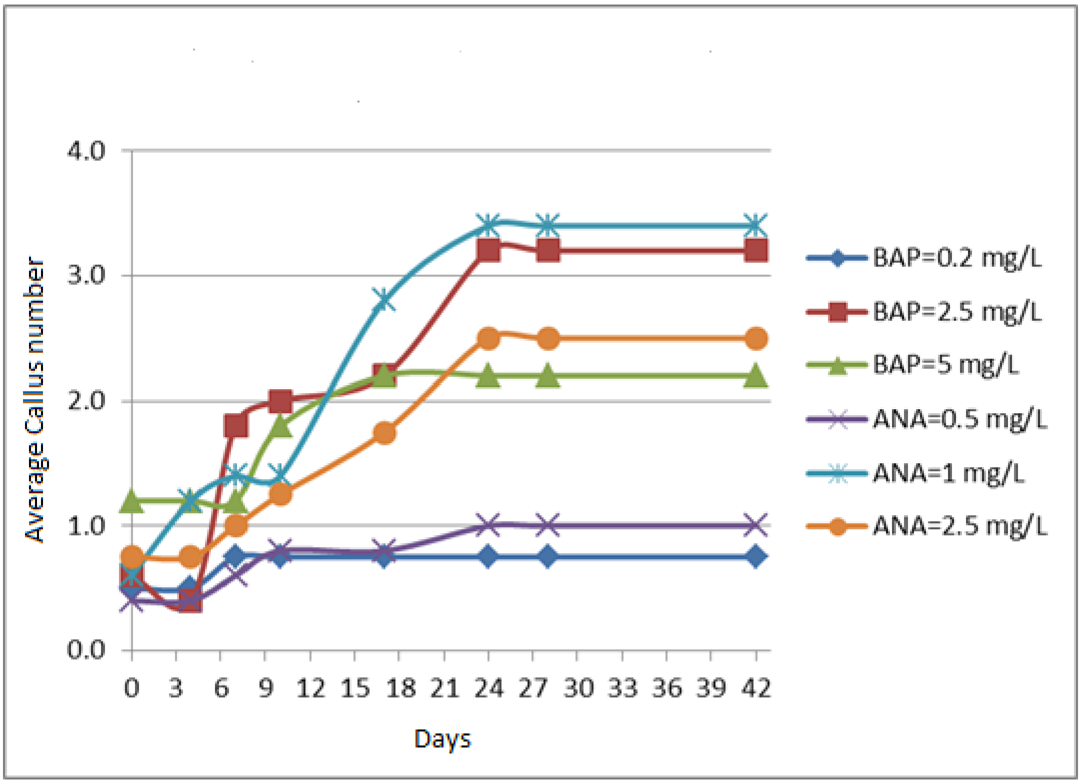

Figure S3. Effect of 6-BAP and NAA on callus induction in *R. officinalis explants* versus days of cultivation.

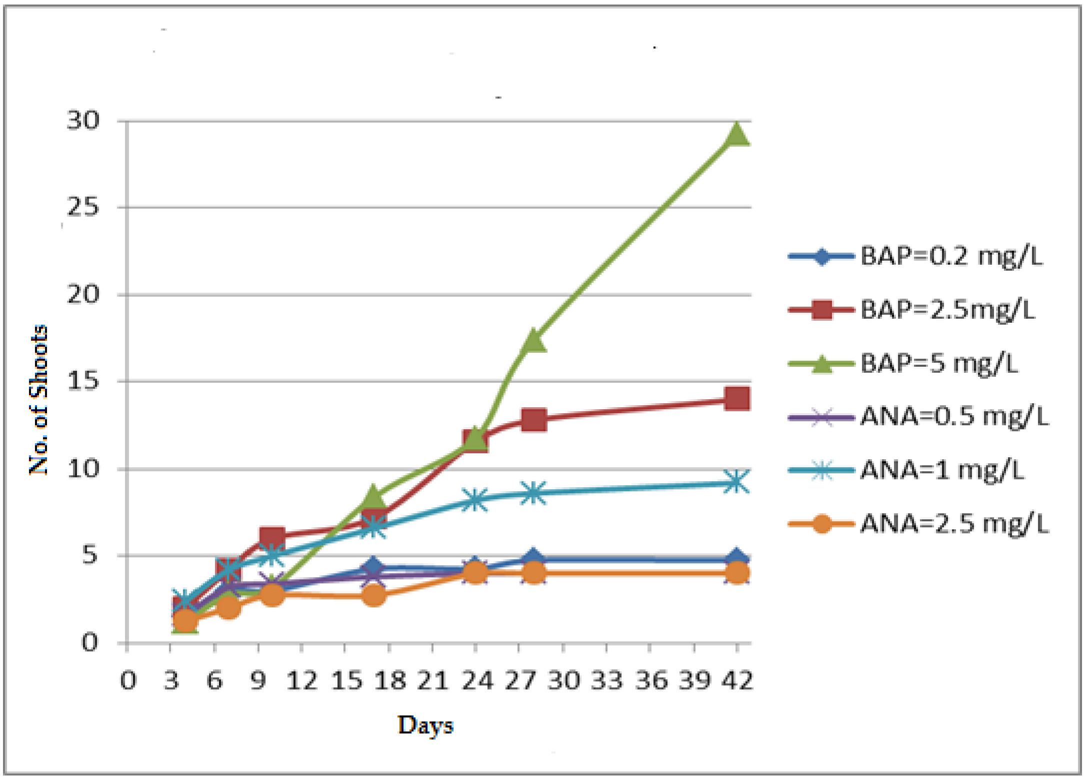

Figure S4. Effect of 6-BAP and NAA on number of shoots in R. officinalis explants versus days of cultivation.

Thin-layer chromatography

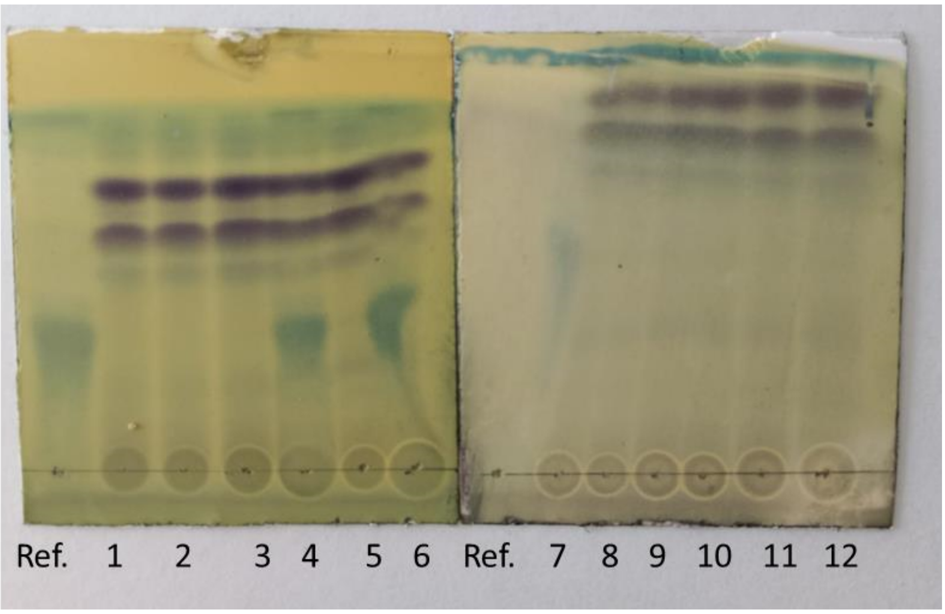

Figure S5. Thin-layer chromatography (TLC, SiO_2) of acetonic extracts of callus samples from fractions 1-12 (Table 1) developed with dichloromethane: acetone (95:5) and revealed with oleum (80% acetic acid, 16% water, 4% sulphuric acid).

HPLC chromatogram

Table S4. Validation parameters for the determination of carnosol by HPLC-DAD.

Validation parameters	Results
**Wavelength (nm)**	293
**Linearity range (µg / mL)**	150-385
**Retention time (min)**	10.7
**Calibration equation**	y= 0.005x + 0.0256
**Correlation coefficient (R^2)**	0.9894
**Standard deviation of the intercept**	3.0×10^{-4}
**Standard deviation of the slope**	0.0256
**Limit of detection (µg/mL)**	3.68
**Limit of quantification (µg/mL)**	12.62

Table S5. Influence of the flow of the mobile on retention time (t_R), dead time (t_0) and retention factors (k′).

Flow (mL/min)	Retention time (min)	Dead time (min)	Retention factors
1.0	14.81	2.513	4.89
1.1	13.44	2.293	4.86
1.2	10.74	2.680	3.01
1.5	11.32	2.147	4.27

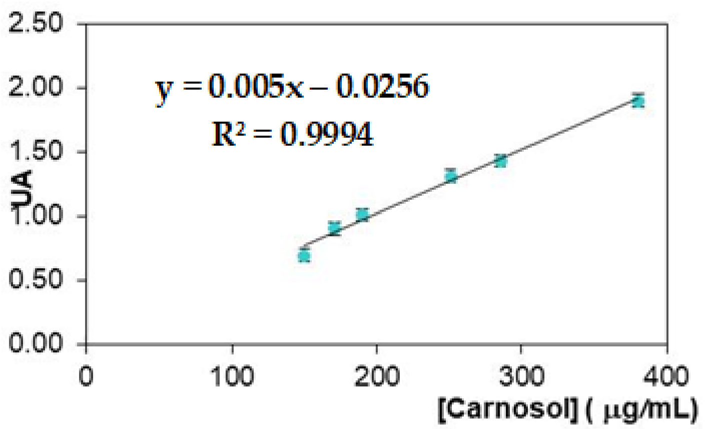

Figure S6. Calibration Curve for carnosol. Six different concentrations of carnosol (n= 3) were prepared from the standard solution. 20 µL of each concentration were injected and response was measured at 293 nm to plot calibration curve.

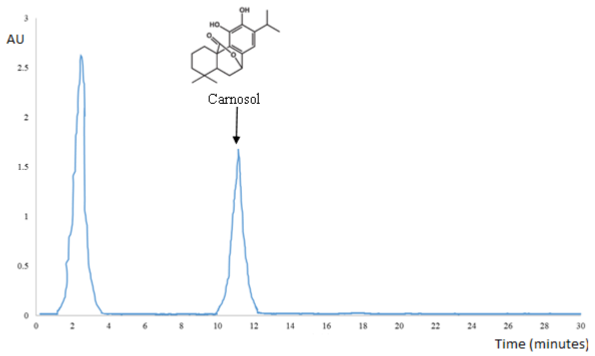

Figure S7. Chromatogram of carnosol at 370 µg mL^{-1}. Chromatography conditions: flow at 1.2 mL min^{-1}; isocratic elution, 60-40% 10 mM formic (pH 3) - CH_3CN (v/v); the injection volume was 20 µL, and the wavelength of the detector was set at 293 nm.

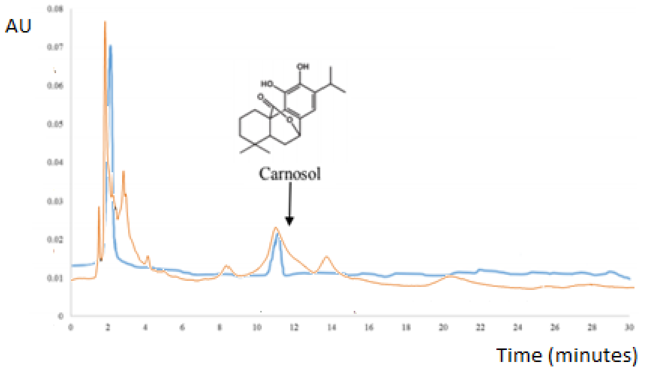

Figure S8. Chromatogram of the Carnosol standard at 220 mg mL^{-1} (blue line) vs chromatogram of a sample of *R. officinalis* acetonic extract (orange line). Chromatography conditions: flow at 1.2 mL min-1; isocratic elution, 60-40% 10 mM formic (pH 3) - CH_3CN (v/v); the injection volume was 20 µL, and the wavelength of the detector was set at 293 nm.

Chromatogram of carnosol at 370 µg mL^{-1}. Chromatography conditions: flow at 1.2 mL min^{-1}; isocratic elution, 60-40% 10 mM formic (pH 3) - CH_3CN (v/v); the injection volume was 20 µL, and the wavelength of the detector was set at 293 nm.

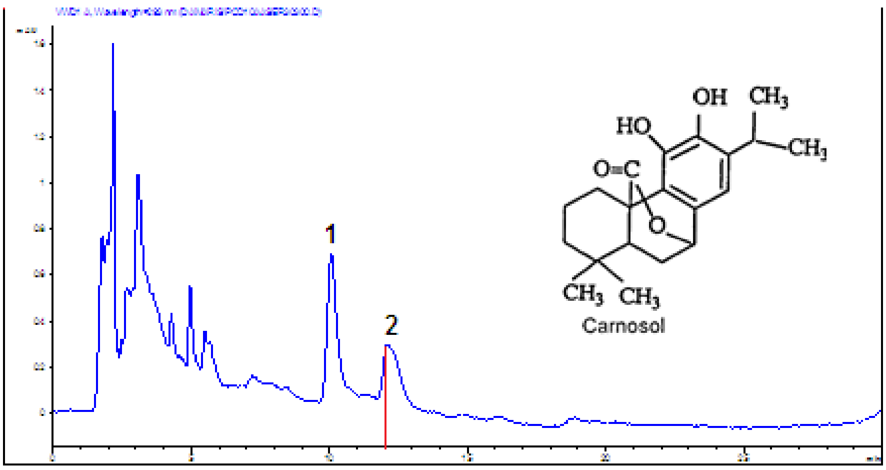

Figure S9. Characteristic HPLC profile of the acetonic extract of a callus sample of *R. officinalis* (media supplemented with 6- BAP; Table 3, entries 1 and 4). Peak 1, carnosol; Peak 2, undefined.

Chromatography conducted using Hypersil ODS C18 (250mm x 4.6 mm, 5 μm particle size) column; flow at 1.2 mL/min; isocratic elution, 60-40% 10 mM formic (pH 3) - CH_3CN (v/v) as mobile phase which was filtered and degassed before analysis; Retention times (r.t) in min correspond to the analysis at 25°C; the injection volume was 20 μL, and the wavelength of the detector was set at 254 and 293 nm.
